# Comparative Usability Evaluation of Three Digital Smile Design Software Tools Using the System Usability Scale

**DOI:** 10.3390/dj13090418

**Published:** 2025-09-12

**Authors:** Andrei Macris, Sergiu Drafta, Ștefania Martiniuc, Alexandru E. Petre

**Affiliations:** 1 Prosthetic Department, Faculty of Stomatology, “Carol Davila” University of Medicine and Pharmacy, 020021 Bucharest, Romania; andrei.macris@umfcd.ro (A.M.); alexandru.petre@umfcd.ro (A.E.P.); 2 Faculty of Stomatology, “Carol Davila” University of Medicine and Pharmacy, 020021 Bucharest, Romania

**Keywords:** Digital Smile Design, System Usability Scale, user experience, aesthetic dentistry

## Abstract

**Background/Objectives:** Digital Smile Design software tools facilitates aesthetic planning and improves communication between clinicians, patients, and dental laboratories. These software tools have been developed to support facial and dental analysis and to assist users in creating an ideal smile integrated with the patient’s appearance. This study aimed to compare the usability of three DSD software tools—Preteeth AI Pro (version 6.0.0), SmileCloud, and Medit Link (version 3.4.3)—using the System Usability Scale. **Methods:** Twenty-three prosthodontists and prosthodontics residents evaluated each tool following a standardized usage protocol. After completing Digital Smile Designs in each application, participants filled out a 10-item System Usability Scale questionnaire (score 0–100). Descriptive statistics were calculated, and intergroup comparisons were performed using one-way ANOVA (*p* < 0.05). **Results:** Mean System Usability Scale scores were 74.24 (Preteeth AI Pro), 80.33 (SmileCloud), and 73.15 (Medit Link). SmileCloud obtained the highest score (A−grade, Curved Grading Scale), indicating “good to very good” usability. No statistical significances were found between the three software tools (F = 1.04, *p* = 0.36). **Conclusions:** All three Digital Smile Design software tools achieved System Usability Scale scores above the usability benchmark of 68, with SmileCloud demonstrating the most favorable user experience. These findings may assist clinicians in selecting intuitive and efficient Digital Smile Design platforms to optimize aesthetic treatment workflows.

## 1. Introduction

Digital Smile Design (DSD) software represents innovative tools in aesthetic dentistry for planning, simulating, and communicating aesthetic outcomes before procedures begin [1]. These platforms integrate photos, intraoral scans, and three-dimensional (3D) models, enabling dentists to create personalized aesthetic plans in collaboration with patients and, occasionally, dental technicians [2]. Although integrating DSD into treatment plans increases patient satisfaction compared to conventional workflows [3], its adoption in dental practices remains limited.

Regarding the existing literature on this subject, there are studies that focus on the interface and aesthetic preferences [1,4], patient satisfaction [3,5], or clinical outcomes [6,7] of DSD tools, rather than the user experience from the clinician’s perspective. There is limited evidence directly comparing DSD applications’ usability in terms of efficiency, learnability, and satisfaction, especially among specialists like prosthodontists who rely heavily on these tools in practice, as recent narrative reviews emphasize applications and benefits without head-to-head usability analyses [2]. This gap highlights the need for systematic, user-centered evaluations using validated instruments such as the System Usability Scale and standardized reporting [8,9].

User Experience (UX) is a critical criterion for selecting and assessing DSD software effectiveness. UX encompasses users’ perceptions, reactions, and satisfaction during software interaction, including usability, interface quality, functional efficiency, and perceived control [10]. A positive UX reduces learning time, enhances practice productivity, and facilitates interdisciplinary communication [11]. Evaluating UX in DSD applications is thus essential for optimizing clinical workflows and improving patient satisfaction. DSD benefits multiple dental fields, including orthodontics, implantology, prosthodontics, and orthognathic surgery, by generating a smile frame—representing the contour of upper and lower teeth—processed to achieve ideal or patient-desired aesthetics [12].

The System Usability Scale (SUS), developed in 1996, is a widely used tool for assessing UX, comprising 10 questions scored from 1 to 5 [13]. Its reliability is supported by alpha coefficients of 0.80–0.95, with content validity ensured by UX experts [14]. SUS reliability is approximately 0.90 [15]. SUS scores are categorized as “fair”, “good”, or “excellent” [16]. This classification based on adjectives was later correlated with a curved rather than an absolute grading scale [17]. For the interpretation of SUS scores obtained in this study we used the Curved Grading Scale (CGS), which ranks scores based on percentiles from over 5000 responses [18]. A score of 68 is considered average (C grade), while scores of 84.1–100 (A+) indicate excellent usability, and scores below 51.6 (F) denote deficient usability. A mean SUS score of at least 80 (A−) reflects above-average UX [17]. However, local samples may differ from international normative groups, requiring cautious interpretation.

Although SUS has not been directly applied to DSD applications, studies on auxiliary dental applications, such as the Salud Electronic Dental Record System (EDR) (SUS score: 77) and Dental Pre-screening System (STO), demonstrate its validity in dental settings [19,20]. Mean SUS scores vary by application type: business-to-business (B2B) software averages 67.6 (C), while internal productivity software (IPS) averages 76.7 (B) [13]. DSD applications serve both B2B (clinic/laboratory communication) and IPS (clinical workflow efficiency) purposes, making these benchmarks relevant. Achieving a SUS score of 80 is a common industry goal, indicating good to very good usability [21].

SUS questions can be adapted with consistent terminology [22]. Responses are scored on a Likert scale (1 = strongly disagree, 5 = strongly agree) [23]. Scores are calculated by subtracting 1 from odd-numbered item responses and subtracting even-numbered item responses from 5, summing the adjusted scores (0–40), and multiplying by 2.5 to yield a 0–100 scale [24]. SUS is adaptable to medical domains, aiding human/computer interaction (HCI) experts in improving design guidelines for enhanced practitioner UX [25].

While individual DSD applications have been described in the literature, to our knowledge, no peer-reviewed studies have compared the usability of multiple DSD soft-ware tools using the SUS framework in a standardized, head-to-head evaluation. This represents a significant gap, given the rapid development of AI-enhanced dental design platforms and the need for evidence-based recommendations to guide practitioners’ soft-ware choices.

The aim of this study was to evaluate and compare the usability of three widely accessible DSD software tools—Preteeth AI Pro (version 6.0.0—https://dentscape.ai/PreteethAIpro), SmileCloud (https://smilecloud.com/), and Medit Link (version 3.4.3)—using the SUS. (all software accessed between 11 June 2025–24 June 2025). Specifically, the objectives were to determine the mean SUS scores for each software, to compare their usability through statistical analysis, and to identify which platform provides the most favorable user experience for prosthodontic applications.

By addressing this gap, the present study seeks to provide clinicians and developers with objective insights into the usability performance of current DSD platforms, supporting more informed adoption decisions and guiding future software development.

## 2. Materials and Methods

Data were collected using a questionnaire based on the SUS model for each software tool. The selected software—Preteeth AI Pro (Dentscape AI, San Francisco, CA, USA - https://dentscape.ai/PreteethAIpro, version 6.0.0), SmileCloud (Smilecloud SRL, Timișoara, Romania—https://smilecloud.com/), and Medit Link version 3.4.3 (Medit Corp., Seoul, Republic of Korea)—were chosen for their accessibility and usability as entry points to DSD (all software have been accessed between 11 June 2025–24 June 2025). Preteeth AI Pro (version 6.0.0) is a mobile application that integrates artificial intelligence, used to quickly assess aesthetic parameters such as the midline, gumline, smile curve and the width/height ratio of the central incisor. Image manipulation consists in bleaching and veneers functions. SmileCloud combines computer-aided design/computer-aided manufacturing (CAD/CAM functions), planning, biometric data, and communication between the clinic and the laboratory, enabling the generation of realistic smiles based on natural tooth shapes selected by artificial intelligence or from biometric libraries. Medit Link (version 3.4.3) is a digital ecosystem that includes a 3D scanning module for an intraoral scanner (IoS) and additional applications such as Smile Design, Ortho Simulation, Model Builder, etc. The Smile Design module within allows digital simulation of the smile based on the patient’s scans and photos and direct integration with the scanner and CAD/CAM software.

Participants included 23 prosthodontists and prosthodontics residents from the Dental Faculty at “Carol Davila” University of Medicine and Pharmacy, Bucharest. Each participant used all three software applications and completed corresponding SUS questionnaires, yielding 69 valid responses. Participants received online training, a written guide with DSD detailed steps and graphic/screenshot exemplification, download and installation instructions, account details, and design flow examples. Two online training sessions were conducted, spaced two weeks apart. Each session included a comprehensive review of all previously presented materials and a step-by-step walkthrough of the protocol for all three software applications. The sessions were delivered in an interactive format, enabling participants to engage in continuous discussion with the trainers. A minimum of two study authors were present during each training session. To ensure uniform testing, participants used identical photographs of one author, eliminating patient consent needs, or Institutional Review Board statement, and uploaded to a shared drive.

Inclusion criteria—eligible participants:

- Prosthodontic specialists aged between 30 and 65 years who reported prior experience with at least one Digital Smile Design (DSD) application and current engagement with digital tools in routine clinical practice (e.g., clinical photography, intraoral scanning, CAD/CAM).
- Prosthodontic residents (years 1–3) aged between 24 and 30 years who reported prior experience with at least one Digital Smile Design (DSD) application and current engagement with digital tools in routine clinical practice (e.g., clinical photography, intraoral scanning, CAD/CAM).
- Participants were required to complete the standardized training sessions.
- Participants were required to complete the standardized workflow, to finalize the three DSD projects, and to submit all 10 SUS items for each software tool.
- Participants must not have any direct commercial involvement with any of the evaluated software developers.
- Additional criterion: complete datasets across all evaluations.

All participants were informed about the aim of this study and voluntarily agreed to complete the usability questionnaire. No identifiable personal or medical data were collected, and therefore formal IRB approval was not required.

The decision to include 23 participants was based on methodological guidelines for usability studies employing the SUS. Previous research has demonstrated that SUS pro-duces reliable and valid results even with relatively small sample sizes, with diminishing returns in additional insights beyond 20–25 participants [13,15]. Each participant evaluated all three software applications, resulting in a total of 69 independent SUS evaluations, which enhances robustness of within-subject comparisons by reducing inter-participant variability.

Demographic data collected from participants included age, gender, and professional status (prosthodontics specialist or first-year resident). These parameters were analyzed descriptively to add context to the usability scores.

The questions used in the adapted questionnaire for the evaluation of DSD applications have been drafted based on those contained in the standard version of the SUS scale. The questions allow flexibility and adaptation to specific domains through minor modifications. The standard version refers to “system”, but replacing this with corresponding terms like “application”, “software”, “product”, “website”, or the exact name of the pro-gram does not appear to have any effect on the results [22]. Any changes made should remain constant across all questions [22] The adapted questions that were used in the questionnaire were the following:

1. I think that I would like to use this software frequently for Digital Smile Design.
2. I found the software unnecessarily complex for the process of making a DSD.
3. I thought the software was easy to use for achieving a complete DSD.
4. I think that I would need the support of a technical person to be able to use the software correctly.
5. I found the various functions in this software were well integrated into the Digital Smile Design workflow.
6. I thought there was too much inconsistency in this software.
7. I would imagine that most dentists would learn to use this software very quickly for the purpose of making a DSD.
8. I found the software very awkward to use (too complicated for the basic DSD functions).
9. I felt very confident using the software to generate an aesthetic design.
10. I needed to learn a lot of things before I could get going with this software.

Data were collected via Google Forms (Google LLC, Mountain View, CA, USA), with responses stored securely and exported to Microsoft Excel (Version 2506 Build 16.0.18925.20076, 64-bit, Microsoft Corp., Redmond, WA, USA) and JASP (version 0.18.3, University of Amsterdam, Amsterdam, The Netherlands) for analysis. Access to complete the form was provided online to all participants. Respondents were informed about the purpose of this research, the anonymous and voluntary nature of their participation, and the manner in which they should answer the questions.

The 10 questions of the SUS questionnaire are divided into odd-numbered items which have a positive meaning and even-numbered items with negative connotations. Each question has a score from 1 to 5, depending on the respondent’s choice. In order to calculate the SUS score, the answers have to be recoded using the following methods:

1. Odd items: subtract 1 from the answer value (answer—1).
2. Even items: the response value is subtracted from 5 (5—response).

The adjusted scores range from 0 to 4, where 4 indicates the most positive perception of the usability.

The recoded responses from all questions are summed, resulting a partial score be-tween 0 and 40. The formula for the total SUS score was Partial score × 2.5.

The final result will be a score between 0 and 100, but it is not a percentage. This score is calculated for each individual application and respondent, and the final SUS score for each software will be the average of all 23 SUS scores obtained.

Data analysis included the following:

1. Descriptive statistics: mean, standard deviation, median, minimum, and maximum SUS scores per software.
2. One-way Analysis of Variance (ANOVA): to test for significant differences between mean scores of the three software applications.
3. Post hoc tests: applied if ANOVA indicated significant differences.
4. Normality of the SUS score distributions for each condition was assessed with the Shapiro–Wilk test (α = 0.05). In the case of minor departures from normality, the robustness of the repeated-measures ANOVA was checked via a nonparametric sensitivity analysis (Friedman test).
5. An exploratory correlation analysis.
6. Statistical significance level: *p* < 0.05.

## 3. Results

**Figure 1:**
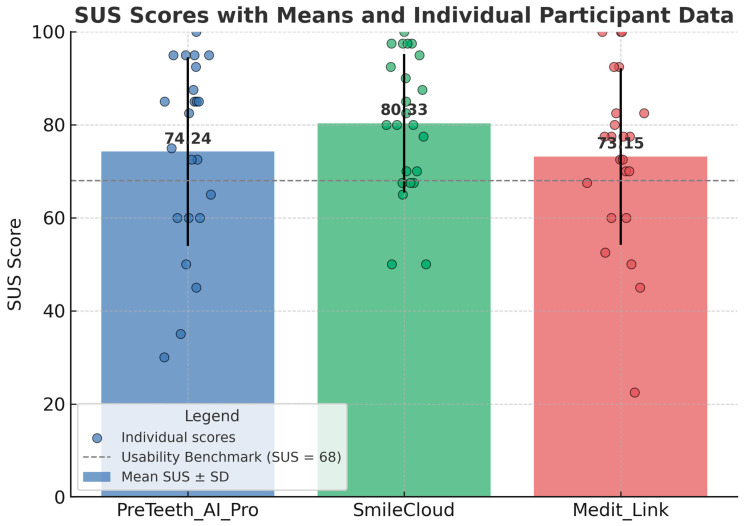
Mean SUS scores with standard deviations (bars) and individual participant scores (dots) for the three evaluated Digital Smile Design software tools (PreTeeth AI Pro, SmileCloud, Medit Link). SmileCloud achieved the highest mean SUS score (80.33), exceeding the usability benchmark of 68, and demonstrated the lowest variability among participants. PreTeeth AI Pro (74.24) and Medit Link (73.15) also exceeded the benchmark but showed greater dispersion in user ratings. The dashed horizontal line represents the SUS usability benchmark (68).

PreTeeth AI Pro (version 6.0.0) obtained a final SUS score of 74.24, being graded as a “B” on the CGS. This corresponds to a fair level of usability. After analyzing all 23 responses, SmileCloud achieved a final SUS score of 80.33 and an “A−” (above average) grade. Medit Link (version 3.4.3) obtained a SUS score of 73.15 and a “B−” grade, also indicating a fair level of usability. In conclusion, among the three DSD software applications, SmileCloud (80.33) is the only one with good to almost very good usability, representing a high level of user satisfaction. PreTeeth AI Pro (version 6.0.0) (74.24) and Medit Link (version 3.4.3) (73.15) had similar scores, both falling within the “fair” category; however, they are above the average benchmark of 68 according to the CGS interpretation system (Table 1 and Figure 1).

Statistical analysis (Table 2) showed the following:

- SmileCloud recorded the highest mean SUS score (M = 80.33; SD = 14.91), indicating a higher perceived usability compared to the other software applications. The 25th (68.75) and 75th (93.75) percentiles suggest a concentration of scores in the higher range. SmileCloud appears to provide the most consistently positive user experience, as reflected by the lowest standard deviation.
- Preteeth AI Pro (version 6.0.0) obtained a mean SUS score of 74.24 (SD = 20.30), with a median of 82.5 and a wide score range (30–100), suggesting greater variability in user perceptions.
- Medit Link (version 3.4.3) achieved a mean SUS score of 73.15 (SD = 18.98), with scores ranging from 22.5 to 100. Although the median is relatively high (77.5), the widest range among all three applications (77.5) indicates a notable dispersion in user experiences.

Before interpreting the ANOVA results, the normality assumption for each dataset was verified. Shapiro–Wilk tests indicated the following: PreTeeth AI Pro (version 6.0.0) W = 0.911, *p* = 0.044; SmileCloud W = 0.926, *p* = 0.091; Medit Link (version 3.4.3.) W = 0.944, *p* = 0.218. Thus, one distribution showed a slight deviation from normality (PreTeeth AI Pro version 6.0.0), whereas the other two met the normality assumption. Given the balanced within-subject design and comparable variances, the repeated-measures ANOVA is considered robust for such a mild deviation. As a sensitivity check, a Friedman test was conducted and yielded consistent results (χ^2^(2) = 1.49, *p* = 0.475), supporting the conclusion that there were no statistically significant differences among the software tools.

In order to determine whether statistically significant differences existed between the mean SUS scores obtained for the software applications (PreTeeth AI Pro version 6.0.0, SmileCloud, and Medit Link version 3.4.3), a one-way ANOVA test was applied. The *p*-value represents the probability that the observed differences between the mean scores occurred by chance. According to statistical convention, a *p*-value < 0.05 indicates that the differences are statistically significant. The F-value is the ratio of the variance between groups to the variance within groups. The higher the F-value, the more likely it is that the differences between groups are real and not attributable to chance.

The results of the test showed an F-value = 1.04 and a significance level of *p* = 0.36. Since the *p*-value = 0.36 > 0.05, there are no statistically significant differences between the mean SUS scores of the three applications. Statistically, this indicates that none of the ap-plications is significantly superior to the others in terms of usability, given the small difference between SUS scores.

A post hoc power analysis was performed using the observed effect size (partial η^2^ = 0.078; Cohen’s f = 0.29) derived from the repeated-measures ANOVA results (F(2, 44) = 1.037; *p* = 0.360). With *n* = 23 participants, α = 0.05, and three conditions, the achieved statistical power was 0.19. This value indicates adequate sensitivity for detecting medium-to-large effects, which aligns with typical objectives of SUS-based comparative studies, but limited sensitivity for detecting small differences in usability.

Comparable SUS-based studies in dentistry and healthcare software usability have employed sample sizes in the range of 15–30 participants [19,20], supporting the adequacy of our sample size for the present study’s objectives. Nonetheless, we acknowledge that larger and more diverse participant groups could increase the generalizability of the findings and improve sensitivity to smaller usability differences (Table 3).

An exploratory correlation analysis was conducted to examine potential associations between participants’ demographic characteristics (gender, professional status, and age interval) and the System Usability Scale (SUS) scores for each evaluated software application. Gender was coded as 0 = female and 1 = male, while professional status was coded as 0 = resident and 1 = specialist. Age interval was converted to an approximate numeric midpoint for correlation purposes (Table 4).

A point-biserial correlation revealed a statistically significant negative association between gender and SUS scores for both PreTeeth AI Pro (version 6.0.0) (r = −0.47, *p* = 0.030) and Medit Link (version 3.4.3) (r = −0.47, *p* = 0.033), indicating that female participants tended to rate these applications more favorably than male participants. No statistically significant correlation be-tween gender and SUS score was observed for SmileCloud (*p* = 0.181) (Table 4).

Professional status and age (which were highly collinear in the present sample) showed a statistically significant negative correlation with SUS scores for SmileCloud (r = −0.56, *p* = 0.009), suggesting that residents tended to assign higher scores than specialists. For PreTeeth AI Pro (version 6.0.0) and Medit Link (version 3.4.3), the associations with professional status and age were not statistically significant (*p* > 0.05).

Overall, the results indicate that gender differences may influence perceived usability for PreTeeth AI Pro (version 6.0.0) and Medit Link (version 3.4.3), while professional experience (status) appears to in-fluence ratings for SmileCloud. However, given the sample size, these findings should be interpreted cautiously and warrant further investigation in larger, more diverse cohorts.

The results synthesis is as follows:

1. The primary outcome was the SUS score for each software tool. Descriptive statistics were computed for each application, including the mean, standard deviation SD, median, minimum, maximum, and interquartile range. The usability benchmark was set at a SUS score of 68, based on the CGS.
2. Given the within-subject design (each of the 23 participants evaluated all three software applications), a one-way repeated-measures Analysis of Variance (ANOVA) was applied to compare the mean SUS scores. Prior to ANOVA, the assumption of normality for each distribution was tested using the Shapiro–Wilk test, and the assumption of sphericity was verified using Mauchly’s test. When sphericity was violated, the Greenhouse–Geisser correction was applied (not required for the present dataset). Effect size was reported as partial eta squared (η^2^_p_), with its corresponding interpretation, and Cohen’s f was calculated.
3. The repeated-measures ANOVA yielded F(2, 44) = 1.037, *p* = 0.360 (two-tailed), partial η^2^ = 0.078, Cohen’s f = 0.29. Exact *p*-values are reported to three decimal places. Ninety-five percent confidence intervals (95% CI) for the mean SUS scores were PreTeeth AI Pro (version 6.0.0) = 65.41–83.06, SmileCloud = 73.96–86.70, and Medit Link (version 3.4.3) = 65.91–80.39 (Table 3).
4. A post hoc statistical power analysis was conducted in JASP using the observed effect size and study parameters (*n* = 23, k = 3, α = 0.05), which indicated an achieved power of 0.19. This confirms adequate sensitivity for detecting medium-to-large effects but limited sensitivity for small effects.

## 4. Discussion

The SUS enabled direct comparison of PreTeeth AI Pro (version 6.0.0), SmileCloud, and Medit Link (version 3.4.3). SmileCloud’s mean score of 80.33 (A−, CGS) reflects high usability, likely due to its intuitive interface and collaborative features [25]. PreTeeth AI Pro (version 6.0.0) (74.24) and Medit Link (version 3.4.3) (73.15) scored above the SUS benchmark of 68 (C) but below SmileCloud, with higher standard deviations suggesting less consistent UX, possibly due to complex interfaces or steeper learning curves.

A closer examination of the individual SUS items offers additional insights into the usability patterns observed. For SmileCloud, higher scores were particularly evident in items related to ease of learning and confidence in using the system, suggesting that its interface and workflow may be more intuitive for first-time users. Conversely, PreTeeth AI Pro (version 6.0.0) and Medit Link (version 3.4.3) received comparatively lower ratings on items addressing the integration of functions and the need for technical support, indicating potential areas for interface refinement and better in-app guidance. These item-level differences highlight that overall, SUS scores, while informative, may mask specific strengths and weaknesses that can guide targeted improvements. For software developers, addressing lower-scoring aspects—such as simplifying complex navigation or enhancing contextual help—could directly improve user satisfaction and adoption. From a clinical perspective, training programs should focus on familiarizing practitioners with features identified as less intuitive, thereby reducing the learning curve and optimizing the clinical benefits of these tools.

When compared to similar investigations on dental software usability, the SUS scores reported in this study are broadly consistent with findings from recent research on digital dental design platforms [9,15,19]. Previous studies evaluating clinical CAD/CAM soft-ware and DSD applications have typically reported mean SUS scores ranging from the mid 60 s to low 80 s, depending on user familiarity and interface complexity [5,15,22]. The above-average ratings obtained for SmileCloud align with reports emphasizing the impact of intuitive design and streamlined workflows on perceived usability [9,17], while the fair ratings of PreTeeth AI Pro (version 6.0.0) and Medit Link (version 3.4.3) correspond to earlier observations that multistep navigation and limited contextual assistance can reduce user satisfaction [15,19]. These parallels suggest that the trends identified in our cohort may be generalizable to other settings and user populations.

Compared to B2B software (mean SUS = 67.6, C) [13], all three applications performed better. As IPS, only SmileCloud exceeded the average of 76.7 (B) [13]. SmileCloud also met the industry goal of a SUS score of 80 [21]. Its advantages include an intuitive visual interface, cloud collaboration, simpler functions, and fewer steps for DSD, potentially enhanced by familiarity among Romanian practitioners [25]. Preteeth AI Pro (version 6.0.0) and Medit Link (version 3.4.3) may require more technical expertise, impacting usability.

When evaluating the applications as business-to-business (B2B) programs that connect clinical practice with the laboratory, their scores can be compared to the literature’s average B2B score of 67.6 (C) [13]. All three applications surpassed this benchmark. However, these applications primarily function as internal productivity software (IPS) used and evaluated by dental practitioners. From this perspective, only SmileCloud exceeds the literature’s average IPS score of 76.7 (B) [13], while the other two applications fall below this average. Regarding other dental software, the mobile application for oral health pre-screening (STO), as noted in a previously mentioned study [20], achieved a mean SUS score of 76.9, which is comparable to the scores of the DSD applications in this study. Concerning the industry goal of achieving a mean SUS score of 80 [21], SmileCloud is the only application in this study to meet this threshold.

This study’s clinical relevance lies in guiding dental professionals toward user-friendly DSD tools, critical as digital workflows gain prominence. It contributes to an underexplored academic area, encouraging systematic evaluation of dental digital tools.

The limitations of this study stem from its sample size and the subjective nature of responses. With 23 responses per application, the sample size, though sufficient for SUS, may not fully represent the broader dental community or account for variations in experience levels, regions, or practice types. Although standardized, the SUS relies on subjective perceptions, and differences in user expectations, technological proficiency, or learning curves may bias results. Additionally, this study’s quantitative approach does not explore why certain applications were deemed more usable, omitting details such as interface layout issues, specific feature strengths or weaknesses, or training needs. The evaluation also excludes critical factors like clinical accuracy, CAD/CAM integration, cost, or support services, all of which influence software selection. However, the findings enhance understanding of how usability affects the adoption and efficiency of digital tools in aesthetic dentistry. As digital workflows become increasingly vital, selecting user-friendly software is essential for improving clinical outcomes, enhancing communication, and boosting patient satisfaction. These results can guide dental professionals in making informed decisions and provide developers with insights to optimize future DSD platforms.

This study did not collect additional demographic parameters such as workplace setting (academic or private practice) or secondary specialties, which may also influence perceptions of software usability. This limitation should be considered when interpreting the findings, as broader demographic data might provide deeper insights into user variability.

One potential source of bias relates to participants’ awareness of the software identity during evaluation. Blinding to software names was not feasible due to the distinctive user interfaces and proprietary branding of each application. To mitigate potential bias, the order of software evaluation was randomized across participants, standardized case materials and tasks were used, and participants were instructed to base their SUS ratings solely on functionality, ease of use, and overall usability.

From a practical standpoint, these results can inform both software selection by clinicians and development priorities for manufacturers. For clinicians, recognizing the relative strengths and weaknesses of each application can guide targeted training, thereby reducing the learning curve and improving integration into daily workflows. For developers, focusing on areas identified as less intuitive—such as function integration, navigation simplification, and real-time user support—could enhance user satisfaction and adoption rates. Future research should build upon these findings by involving a larger and more diverse pool of participants, extending evaluations to include long-term use, and incorporating objective performance metrics such as task completion time or error rates, alongside subjective SUS ratings.

Future research should include larger, more diverse participant groups to improve the generalizability of findings and longitudinal studies to evaluate usability over time. Incorporating qualitative feedback could offer deeper insights into user experience, while examining correlations between usability and clinical outcomes may reveal practical benefits. Additionally, future studies should assess the interoperability of DSD applications with other digital dental systems in real-world practice.

## 5. Conclusions

Within the limitations of this study, SmileCloud obtained the highest perceived usability score, while PreTeeth AI Pro (version 6.0.0) and Medit Link (version 3.4.3) achieved comparable, fair-level SUS scores, both exceeding the average benchmark. Although statistical analysis revealed no significant differences among the three software applications, item-level findings pointed to specific areas for improvement, particularly in function integration and user support.

These results may guide clinicians in selecting appropriate DSD tools and inform developers in implementing targeted refinements.

Future studies should involve larger and more diverse participant groups, assess long-term use, and combine subjective usability measures with objective performance metrics for a comprehensive evaluation.

## Figures and Tables

**Table 1:** Summary of System Usability Scale (SUS) scores and corresponding CGS grades for three dental software applications.

Software	Mean SUS Score	CGS Grade	Interpretation
PreTeeth AI Pro	74.72	B	Fair
SmileCloud	80.33	A−	Good ^1^
Medit Link	73.15	B−	Fair

SUS scores range from 0 to 100, with higher scores indicating better usability. CGS grades reflect the usability interpretation based on the SUS score, where A− (80–84) indicates “Good” usability, B (70–79) indicates “Fair” usability, and B− (68–70) indicates marginally “Fair” usability. The “Good ^1^” notation for SmileCloud indicates the highest usability rating among the tested software.

**Table 2:** Descriptive statistics of System Usability Scale (SUS) scores for three dental software tools.

Software	*n*	SUS Mean Score	Standard Deviation	Min	Max	Median	25th Percentile	75th Percentile
PreTeeth AI Pro	23	74.24	20.30	30	100	82.5	60.00	90.00
SmileCloud	23	80.33	14.91	50	100	80.0	68.75	93.75 ^1^
Medit Link	23	73.15	18.98	22.5	100	77.5	63.75	82.50

SUS scores range from 0 to 100, with higher scores indicating better usability. “*n*” represents the number of participants who evaluated each software. The standard deviation reflects the variability of SUS scores. The 25th and 75th percentiles indicate the lower and upper quartiles of the score distribution, respectively. The “^1^” notation for SmileCloud’s 75th percentile clarifies that the value (93.75) is the highest among the tested software for this metric.

**Table 3:** SUS scores for each participant and descriptive statistics for the three evaluated Digital Smile Design tools (PreTeeth AI Pro, SmileCloud, and Medit Link).

Software	PreTeeth AI Pro	SmileCloud	Medit Link
	87.5	50	50
	65	82.5	22.5
	30	50	52.5
	95	70	80
	60	70	70
	95	90	77.5
	92.5	97.5	100
	85	87.5	72.5
	82.5	100	100
	95	97.5	100
	72.5	80	92.5
	60	65	82.5
	85	85	82.5
	85	97.5	60
	50	92.5	60
	72.5	67.5	77.5
	35	80	45
	75	67.5	70
	45	80	72.5
	60	67.5	77.5
	95	95	67.5
	85	77.5	77.5
	100	97.5	92.5
SUS mean score	74.24	80.33 ^1^	73.15

SUS = System Usability Scale. Scores are reported for each participant (rows 1–23). The final row presents the mean SUS score for each software application. A SUS score ≥ 68 indicates usability above the benchmark defined by the Curved Grading Scale (CGS). Higher scores indicate better perceived usability. ^1^ SmileCloud obtained the highest SUS score.

**Table 4:** Correlation coefficients (r) and *p*-values for associations between demographic variables (gender, professional status, and age) and SUS scores for evaluated software applications.

	Software	Gender r	Gender *p*	Status r	Status *p*	Age r	Age *p*
0	PreTeeth AI Pro	−0.74	0.030 **	−0.19	0.421	−0.19	0.421
1	SmileCloud	−0.30	0.181	−0.56	0.009 ***	−0.56	0.009 ***
2	Medit Link	−0.47	0.033 **	−0.27	0.229	−0.27	0.229

SUS = System Usability Scale. Gender coded as 0 = female, 1 = male; professional status coded as 0 = resident, 1 = specialist; age interval converted to midpoint value. Significant negative correlations were found between gender and SUS scores for PreTeeth AI Pro (r = −0.47, *p* = 0.030) and Medit Link (r = −0.47, *p* = 0.033), indicating higher ratings from female participants. Professional status and age were significantly negatively correlated with SmileCloud scores (r = −0.56, *p* = 0.009), suggesting that residents tended to give higher ratings than specialists. No other associations reached statistical significance (*p* > 0.05); ** *p* < 0.05 (statistically significant); *** *p* < 0.01 (statistically significant).

## Data Availability

The data that support the findings of this study are available from the corresponding author upon reasonable request.
